# Histamine H2-Blocker and Proton Pump Inhibitor Use and the Risk of Pneumonia in Acute Stroke: A Retrospective Analysis on Susceptible Patients

**DOI:** 10.1371/journal.pone.0169300

**Published:** 2017-01-13

**Authors:** Nobuhiko Arai, Tomoki Nakamizo, Hikaru Ihara, Takashi Koide, Akiyoshi Nakamura, Masanao Tabuse, Hiromichi Miyazaki

**Affiliations:** 1 Department of Neurological Surgery, Hiratsuka City Hospital, Hiratsuka City, Kanagawa, Japan; 2 Department of Neurology, Hiratsuka City Hospital, Hiratsuka City, Kanagawa, Japan; Queen's University Belfast, UNITED KINGDOM

## Abstract

**Background:**

Although histamine H2-blockers (H2B) and proton pump inhibitors (PPI) are used commonly to prevent gastrointestinal bleeding in acute stroke, they are implicated in the increased risk of pneumonia in other disease populations. In acute stroke, the presence of distinctive risk factors of pneumonia, including dysphagia and impaired consciousness, makes inclusive analysis vulnerable to confounding. Our aim was to assess whether acid-suppressive drugs increase pneumonia in acute stroke in a population controlled for confounding.

**Methods:**

We analyzed acute stroke patients admitted to a tertiary care hospital. To minimize confounding, we only included subjects who could not feed orally during 14 days of hospitalization. Exposure was defined as H2B or PPI, given in days; the outcome was development of pneumonia within this period. The incidence was calculated from the total number of pneumonias divided by the sum of person-days at risk. We additionally performed multivariate Poisson regression and propensity score analyses, although the restriction largely eliminated the need for multivariate adjustment.

**Results:**

A total of 132 pneumonias occurred in 3582 person-days. The incidence was 3.69%/person-day (95% confidence interval (CI); 3.03–4.37%/day). All subjects had dysphagia. Stroke severity and consciousness disturbances were well-balanced between the groups exposed to H2B, PPI, or none. The relative risk (RR) compared with the unexposed was 1.22 in H2B (95%CI; 0.83–1.81) and 2.07 in PPI (95% CI; 1.13–3.62). The RR of PPI compared with H2B was 1.69 (95%CI; 0.95–2.89). In multivariate regression analysis, the RRs of H2B and PPI were 1.24 (95% CI; 0.85–1.81) and 2.00 (95% CI; 1.12–3.57), respectively; in propensity score analyses they were 1.17 (95% CI; 0.89–1.54) and 2.13 (95% CI; 1.60–2.84).

**Conclusions:**

The results of this study suggested that prophylactic acid-suppressive therapy with PPI may have to be avoided in acute stroke patients susceptible to pneumonia.

## Introduction

Pneumonia, a common complication of stroke, is associated with mortality [1–3] and morbidity [2, 3]. In addition, gastrointestinal bleeding (GIB) caused by stress-related mucosal damage is a life-threatening stroke complication [4–6]. To prevent GIB in acute stroke patients, acid-suppressive medications such as histamine H2-blockers (H2B) or proton pump inhibitors (PPI) are commonly administered [7–9], although little evidence supports such preventive therapy.

On the contrary, acid-suppressive drugs are implicated in the increased risk of infections by raising gastric pH and thereby promoting bacterial growth. An association between acid-suppressive drugs and pneumonia has been reported in critical care [10, 11] and in hospital- and community-acquired pneumonia [10, 12, 13]. A similar association may exist between acid-suppressive drug use and pneumonia in acute stroke; therefore, physicians should be cautious about the preventive use of acid-suppressive drugs. However, because patients with acute stroke frequently have distinctive symptoms that strongly predispose to pneumonia, such as dysphagia and impaired consciousness [2], the results in other populations may not necessarily apply to acute stroke patients.

Recently, three studies investigated this association in acute stroke patients but showed inconsistent results [7–9]. Herzig et al [7] found that acid-suppressive medications, especially PPI, were significantly associated with pneumonia. Another study found that PPI were associated with increased risk of pneumonia in chronic stroke, but not in acute stroke [8]. The remaining study [9] found similar prevalence of pneumonia between patients receiving PPI and those receiving H2B. That study, however, did not compare with unexposed controls, and was limited by inadequate information on individual clinical courses. Furthermore, the above studies included all patients presenting with acute stroke. Because patients with acute stroke are a heterogeneous population [2, 3, 14–18], the previous studies included patients with virtually no risk of pneumonia, as well as those with a high risk, according to recently proposed risk scores [14–17]. Although this heterogeneity was addressed using multivariate regression models, such inclusive analysis can lead to residual confounding, depending on model specification [19].

To investigate the relationship between acid-suppressive drugs and pneumonia in acute stroke, we conducted a retrospective study. To minimize confounding [20], we restricted the subjects to those who were susceptible to pneumonia.

## Methods

### Study population

We conducted a retrospective observational study on acute stroke patients who were admitted to a tertiary care hospital in Hiratsuka, Japan from January 1, 2006 through January 1, 2016. This study period was chosen according to a preliminary analysis of 297 person-days, assuming a baseline incidence of 0.43%/person/day, which was the incidence of those receiving no therapy in the preliminary analysis. We included sufficient subjects to obtain >90% and >80% power to detect a two-fold risk increase, compared with no therapy, associated with H2B and PPI, respectively. This study was approved by the Hiratsuka City Hospital Ethical Committee and was granted a waiver of informed consent. The diagnosis of stroke was defined as the onset of neurological symptoms within 48 hours of admission confirmed by the presence of ischemic or hemorrhagic brain lesions by neuroimaging. We employed restriction, an extremely effective way to prevent or minimize confounding [20]. To restrict the subjects so that they were homogeneous with respect to important risk factors of pneumonia, we included only those who could not feed orally (enteral feeding was permitted) for 14 days or more after admission. We chose this period because we expected that it would reliably screen for patients with dysphagia, severe neurological deficits, or impaired consciousness, all outstanding risk factors of pneumonia [2, 14–18]. Moreover, such patients would require tube feeding, also a potent risk factor of pneumonia [18, 21, 22]. We then excluded patients who met any of the following criteria: (1) younger than 18; (2) received a prescription of H2B or PPI before admission; (3) presented pneumonia at admission; (4) under neuro-critical care including surgery and/or mechanical ventilation; (5) had incomplete medical data.

### Exposure to acid-suppressive drugs, outcomes, and incidence of pneumonia

Patients were followed for 14 days from their admission, and their exposure status and the development of pneumonia was recorded. Exposure to H2B and PPI was confirmed by reading patient medical charts. We recorded each patient’s daily exposure and measured the days at risk. Then, they were added to calculate the sum of person-days at risk for each exposure. If patients’ exposure status changed (e.g. from H2B to PPI), days at risk were measured separately for each exposure, and added to the respective sum. Because such crossover may raise concern about misclassification, we performed an additional analysis of subjects excluding those who received both drugs. The choice of drugs was at the discretion of the attending physicians. The days during which antibiotics were administered for any purpose, including for the treatment of the pneumonias, were excluded from person-days at risk. Because prophylactic antibiotics may not effectively prevent pneumonia in acute stroke [23], we also included the days during which antibiotics were administered for other infections in a separate analysis. The outcome was the diagnosis of pneumonia within two weeks after admission, according to the Centers for Disease Control and Prevention criteria [24]. The incidence of pneumonia was calculated from the total number of pneumonias divided by the sum of person-days at risk. Although we employed restriction to diminish the need for multivariate analyses, we additionally performed multivariate Poisson regression and propensity score (PS) analyses, to adjusted for possible confounders, if any.

### Characteristics and comorbidities

The following patient characteristics and comorbidities were documented in medical charts: age, sex, initial NIH stroke scale (NIHSS) score, initial Glasgow coma scale (GCS) score, chronic obstructive pulmonary disease (COPD), diabetes mellitus (DM), dementia, prior pneumonia, coronary artery disease, congestive heart failure, alcohol, smoking, cancer, peripheral artery disease, and peptic ulcer disease. The scores of the GCS and NIHSS were measured at the first examination by neurologists. NIHSS scores were assigned retrospectively according to validated algorithms if they were missing [25].

### Statistical analysis

The Wilcoxon signed-rank test and Fisher’s exact tests were used to compare continuous/ordinal variables and categorical variables, respectively. The incidence of pneumonia and relative risk (RR) were estimated assuming a Poisson distribution. Multivariate Poisson regression analyses were performed using a model including the following covariates: age, sex, initial NIHSS, initial GCS, and history of COPD, DM, congestive heart failure, alcohol, and smoking; all of which are components of at least one of the proposed risk scores for pneumonia in acute stroke [14–17]. PS analysis was performed by calculating the PS with generalized boosted models followed by inverse probability weighting [26]. All covariates were included in the model to calculate PS. The sample size required to obtain sufficient powers to detect assumed RR were estimated by Monte-Carlo simulation. A two-sided type I error of < 0.05 was chosen to indicate statistical significance for all comparisons. All analyses were performed by using the statistical programming environment R [27].

## Results

**Fig 1:**
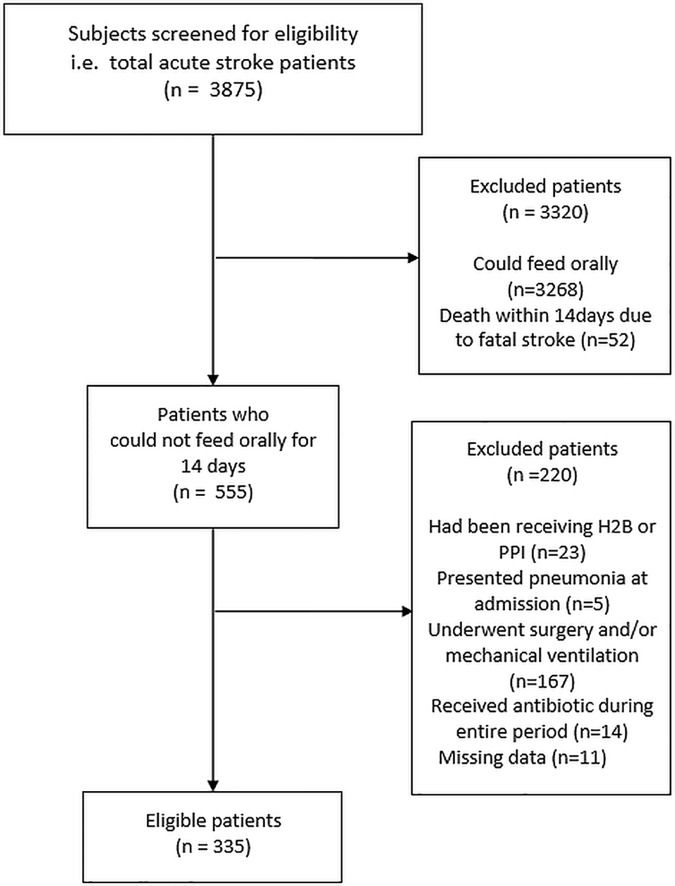
Patient selection.

A total of 3875 patients were admitted with acute stroke from January 1, 2006 through January 1, 2016. Among those, 555 met the inclusion criteria. After excluding patients who had been receiving acid-suppressive medications before admission (n = 23), presented pneumonia at admission (n = 5), underwent surgery and/or mechanical ventilation during the first two weeks of hospitalization (n = 167), received antibiotics during the entire two weeks after admission (n = 14), or were missing data (n = 11), there were 335 final subjects (Fig 1).

Of the 335 eligible patients, 252 had ischemic stroke and 83 had intracerebral hemorrhage. Tube feeding was administered at least once for all our patients. Overall, the median age was 82 years (interquartile range (IQR), 74–88 years) and 160 (48%) were men. The median (IQR) of the NIHSS and GCS scores were 15 (11–24) and 11 (9–14), respectively. In total, 206 patients (61.5%) received acid-suppressive drugs; 174 (51.9%) received only H2B, 26 (7.8%) received only PPI. Six (1.8%) received each drug during separate periods; none received both drugs simultaneously. The administered H2B was famotidine (20–40 mg/day); the PPI was omeprazole (20–40 mg/day). Table 1 lists the demographic characteristics and comorbidities of the subjects. Patients exposed to H2B were not significantly different from those who were unexposed. In contrast, patients exposed to PPI were significantly younger, and had a higher prevalence of DM, coronary artery disease, alcohol, and smoking.

**Table 1:** Patient Characteristics.

Characteristic	None (n = 153)	H2B (n = 180)	*P* value (vs. None)	PPI (n = 32)	*P* value (vs. None)
Age, median (IQR)	83 (76–89)	82 (74–88)	0.087	76.5 (70–84)	0.004
Male, n (%)	64 (41.8)	79 (43.9)	0.740	17 (53.1)	0.248
Initial NIHSS score, median (IQR)	16 (11–24)	15 (11–23)	0.419	12 (11–20)	0.143
Initial GCS score, median (IQR)	11 (9–13)	12 (9–14)	0.220	13 (10–14)	0.068
Comorbidities, n (%)					
COPD	3 (2.0)	4 (2.2)	1	1 (3.1)	0.535
Diabetes mellitus	22 (14.4)	28 (15.6)	0.878	10 (31.3)	0.037
Dementia	22 (14.4)	21 (11.7)	0.514	4 (12.5)	1
Prior pneumonia	3 (2.0)	9 (5.0)	0.155	0 (0)	1
Coronary artery disease	13 (8.5)	16 (8.9)	1	8 (25.0)	0.013
Congestive heart failure	26 (17.0)	41 (22.8)	0.218	9 (28.1)	0.145
Alcohol	18 (11.8)	27 (15.0)	0.424	12 (37.5)	0.001
Smoking	22 (14.4)	34 (18.9)	0.305	11 (34.4)	0.011
Cancer	16 (10.5)	17 (9.4)	0.854	2 (6.3)	0.743
Peripheral artery disease	2 (1.3)	10 (5.6)	0.043	2 (6.3)	0.139
Peptic ulcer disease	6 (3.9)	6 (3.3)	0.778	2 (6.3)	0.629

H2B, histamine H2-blockers; PPI, proton pump inhibitors; IQR, interquartile range; NIHSS, NIH stroke scale; GCS, Glasgow coma scale; COPD, chronic obstructive pulmonary disease.

The total person-day at risk was 3582, in which 132 pneumonias occurred at the rate of 3.69%/day (95% confidence interval (CI); 3.03–4.37%/day). The prevalence was 39.4% (95% CI; 34.2–44.9%). The incidence of pneumonia in the exposed to PPI group was significantly higher than in the unexposed group while that in the exposed to H2B was not (Table 2). The incidence in the exposed to PPI group was nearly 1.7 times higher than that in the exposed to H2B group, although the 95% CIs crossed 1 slightly (Table 2). The days when pneumonia developed were not significantly different between the exposures (S1 Table).

**Table 2:** Incidence and Relative Risk.

Exposure	Pneumonia	Person-days	Incidence (95% CI)	RR (95% CI)
None	47	1521	3.10% (2.27–4.44)	ref
H2B	67	1779	3.77% (2.92–4.78)	1.22 (0.83–1.81) (vs. None)
PPI	18	282	6.38% (3.78–10.1)	2.07 (1.13–3.62) (vs. None)
				1.69 (0.95–2.89) (vs. H2B)

H2B, histamine H2-blockers; PPI, proton pump inhibitors; CI, confidence interval; RR, relative risk; ref, reference

The RRs of the PPI and H2B patients obtained by multivariate regression modeling and by PS analysis (Table 3) were similar, and in agreement with univariate analysis; this consistency suggests that the co-variables included in our models were not major confounders. The c-statistics of the models to calculate PS for PPI and H2B were 0.92 and 0.85, respectively. After inverse probability weighting, all covariates were balanced since differences of standardized means were less than 0.25 for all of them. When subjects outside the common support region were excluded, similar results were obtained (HR 1.08 (95% CI; 0.66–1.79) for H2B, and HR 2.15 (95% CI; 1.38–3.35) for PPI, respectively).

**Table 3:** Multivariate Regression and Propensity Score Analyses.

	RR of H2B (95% CI)	RR of PPI (95% CI)
Multivariate regression	1.24 (0.85–1.81)	2.00 (1.12–3.57)
Propensity score	1.17 (0.89–1.54)	2.13 (1.60–2.84)

H2B, histamine H2-blockers; PPI, proton pump inhibitors; RR, relative risk; CI, confidence interval.

Even when the subjects who were given both H2B and PPI were excluded, the results were similar (S2 Table). In addition, when the days during which antibiotics were administered for infections other than pneumonia were included into the person-days at risk, similar results were obtained (S3 Table).

## Discussion

In this study, we investigated patients with acute stroke who were outside critical care and susceptible to pneumonia, and found that PPI were significantly associated with an increased risk of pneumonia while H2B were not.

The nonsignificant association between H2B and pneumonia demonstrated by our study agrees with previous studies [7, 8], which failed to find a significant association between H2B and pneumonia in acute stroke, although the power was insufficient in one study [7], and not described in the other [8]. Our study was designed with sufficient power to detect a two-fold increase in pneumonia by H2B, and the upper limit of the 95% CI of the RR was about 1.8. Therefore, although there remains a possibility that H2B increases pneumonia risk in acute stroke patients to an extent similar to that observed in critical care [10, 11], our study indicates that H2B use is unlikely to increase pneumonia risk strongly in acute stroke patients.

On the other hand, the use of PPI was associated with over twice the risk of pneumonia compared with unexposed patients. This is in line with the study by J. Herzig et al [7], which demonstrated that the unadjusted and adjusted ORs of PPI for pneumonia were significantly elevated in acute stroke patients to 6.2 and 2.7, respectively. In their study, acid-suppressive drugs were more likely to be prescribed to those who had impaired consciousness and severe dysphagia, both of which are important risk factors of pneumonia in acute stroke patients [2, 14, 15, 17, 18]. Although this confounding was adjusted by multivariate analysis, there might have been some residual confounding. In our study, which was designed to be less vulnerable to confounding, the unadjusted and adjusted RRs (2.07 and 2.00, respectively) were comparable to the adjusted OR in the Herzig study [7]. The similarity in results indicates that use of PPI is indeed associated with an increased risk of pneumonia in acute stroke patients, as observed in other disease populations [10, 12, 13]. Nonetheless, such findings apparently disagree with a study using national insurance claims data in Taiwan [8] that failed to demonstrate the association between PPI and pneumonia in acute stroke patients. The negative result may be due to differences in the definition of exposure, which was at least one use of PPI use during the follow-up. This definition would have underestimated the incidence of pneumonia in the exposed because it included minimally treated patients in the exposed group. Another possible explanation may simply be insufficient power; indeed, in the Taiwanese study, PPI were significantly associated with increased incidence of pneumonia in chronic stroke. The increased risk of pneumonia with use of PPI, but not with H2B, has also been shown in other disease populations [12, 13]. Because PPI may increase pneumonia in acute stroke, physicians should pay great attention to the risks and benefits of PPI when considering their use. In contrast, H2B may be used relatively safely.

In this study, we aimed to minimize confounding by restricting subjects to those who could not feed orally for 14 days or more after admission. This restriction successfully identified susceptible patients, as pneumonia developed in 39% of our patients, a much higher prevalence compared with previous studies [1–3, 28]. This susceptibility probably stems from the presence of four potent risk factors of pneumonia: dysphagia [2, 14, 15, 17 18], stroke severity [2, 14–18], impaired consciousness [15, 17], and tube feeding [18, 21, 22]. All of these factors were balanced between groups in this study, the homogeneity that reduces the possibility of confounding. In addition, similar RRs obtained by univariate, multivariate regression, and PS analyses further suggest that confounding by these identified factors was minimal.

On the other hand, this study has some limitations. First, because our subjects comprised only part of the total acute stroke patients, the results of this study may not be applicable to acute stroke in general. Nevertheless, even if limited, we believe that the results are still clinically relevant because in acute stroke, a substantial fraction of pneumonia cases is expected to arise from patients like our subjects, as inferred from its very high prevalence in this study. In addition, it has been shown that pneumonia [2, 14–18] and GIB [4–6] in acute stroke have common risk factors such as severity and impaired consciousness, and tend to occur in the same patient [28]. This accumulation of pneumonia and GIB in severe patients implies that physicians should consider the risks and benefits of PPI or H2B mainly when they treat patients similar to those presented in this study.

Second, although our study was controlled for confounding, it might have been predisposed to selection bias. Specifically, if a substantial fraction of the patients had been included because of pneumonia, not because of dysphagia or severity, some selection bias could be introduced. However, we believe this bias was minimal for the following reasons: 1) pneumonia generally accumulates in patients with dysphagia and/or with more-than-moderate severity [14–17, 28]; 2) dysphagia in stroke patients typically continues for two weeks after onset, making patients susceptible to pneumonia during the entire period [29, 30]. These observations suggest that most of the patients who developed pneumonia would have been unable to feed orally during the study period even if they had not developed pneumonia. If selection bias were minimal, then our study would have been comparable to a retrospective cohort study.

The third limitation of our study is the exclusion of patients who died within 14 days. They comprised 52 (1.3%) of all strokes (Fig 1), and all of them died of fatal stroke. We excluded such patients for two reasons: 1) they were usually given a “Do Not Resuscitate” order, which might have led to a less rigorous diagnostic attempt of pneumonia; 2) the mortality and morbidity of such patients depends more on stroke than on pneumonia. Although we believe that the exclusion of those patients is reasonable from a practical perspective, it may limit generalizability of this study.

Another limitation of our study is that we could not obtain reliable information about pre-stroke dependence, one of the reported risk factors of pneumonia in acute stroke [2, 14–16], although we measured other important previously reported risk factors.

In conclusion, this study demonstrated that use of PPI was associated with pneumonia in acute stroke patients susceptible to pneumonia while use of H2B was not. Our results suggest that, for physicians considering prophylactic acid-suppressive therapy in acute stroke patients, PPI may have to be avoided in those at high risk for pneumonia.

## Supporting Information

S1 Table Days to pneumonia development. The median and range are presented.
(DOCX)

S2 Table Analyses of subjects excluding those who received both H2B and PPI. Relative risks of H2B and PPI and their 95% confidence intervals are shown.
(DOCX)

S3 Table Analyses including days during which antibiotics were administered. Relative risks of H2B and PPI and their 95% confidence intervals are shown.
(DOCX)
