# Supplementary material for: Histamine H2-Blocker and Proton Pump Inhibitor Use and the Risk of Pneumonia in Acute Stroke: A Retrospective Analysis on Susceptible Patients
Source: PLoS One. 2017 Jan 13;12(1):e0169300. doi: 10.1371/journal.pone.0169300 (PMC5234823; doi:10.1371/journal.pone.0169300)
Supplement: S1 Table — The median and range are presented. (DOCX) [file pone.0169300.s001.docx]

| Exposure | Days from the admission | Days from the first exposure |
| --- | --- | --- |
| None | 7 (1–14) | 7 (1–14) |
| H2B | 6 (1–14) | 5 (1–14) |
| PPI | 9 (1–14) | 8.5 (1–14) |
| P value  (Kruskal-Wallis test) | 0.407 | 0.255 |
