# Supplementary material for: Histamine H2-Blocker and Proton Pump Inhibitor Use and the Risk of Pneumonia in Acute Stroke: A Retrospective Analysis on Susceptible Patients
Source: PLoS One. 2017 Jan 13;12(1):e0169300. doi: 10.1371/journal.pone.0169300 (PMC5234823; doi:10.1371/journal.pone.0169300)
Supplement: S2 Table — Relative risks of H2B and PPI and their 95% confidence intervals are shown. (DOCX) [file pone.0169300.s002.docx]

|  | H2B vs None | PPI vs None | PPI vs H2B |
| --- | --- | --- | --- |
| Univariate | 1.24 (0.83–1.84) | 2.03 (1.07–3.66) | 1.65 (0.89–2.88) |
| Multivariate regression | 1.24 (0.85–1.83) | 1.90 (1.03–3.50) |  |
| Propensity score | 1.20 (0.91–1.59) | 2.08 (1.60–2.72) |  |

H2B, histamine H2-blockers; PPI, proton pump inhibitors.
