# Supplementary material for: Histamine H2-Blocker and Proton Pump Inhibitor Use and the Risk of Pneumonia in Acute Stroke: A Retrospective Analysis on Susceptible Patients
Source: PLoS One. 2017 Jan 13;12(1):e0169300. doi: 10.1371/journal.pone.0169300 (PMC5234823; doi:10.1371/journal.pone.0169300)
Supplement: S3 Table — Relative risks of H2B and PPI and their 95% confidence intervals are shown. (DOCX) [file pone.0169300.s003.docx]

|  | H2B vs None | PPI vs None | PPI vs H2B |
| --- | --- | --- | --- |
| Univariate | 1.24 (0.84–1.84) | 2.17 (1.19–3.81) | 1.75 (0.98–2.98) |
| Multivariate regression | 1.26 (0.86–1.84) | 2.12 (1.19–3.78) |  |
| Propensity score | 1.21 (0.92–1.58) | 2.28 (1.71–3.03) |  |

H2B, histamine H2-blockers; PPI, proton pump inhibitors.
